# Phosphoproteomic profiling of feline mammary carcinoma: Insights into tumor grading and potential therapeutic targets

**DOI:** 10.1371/journal.pone.0330520

**Published:** 2025-08-21

**Authors:** Pruettha Aruvornlop, Sekkarin Ploypetch, Walasinee Sakcamduang, Sirintra Sirivisoot, Tanit Kasantikul, Sittiruk Roytrakul, Narumon Phaonakrop, Nlin Arya

**Affiliations:** 1 Department of Pre-clinic and Applied Animal Science, Faculty of Veterinary Science, Mahidol University, Nakhon Pathom, Thailand; 2 Department of Clinical Sciences and Public Health, Faculty of Veterinary Science, Mahidol University, Nakhon Pathom, Thailand; 3 Center of Excellence in Companion Animal Cancer, Department of Pathology, Faculty of Veterinary Science, Chulalongkorn University, Bangkok, Thailand; 4 Veterinary Diagnostic Laboratory, Michigan State University, Lansing, Michigan, United States of America; 5 National Center for Genetic Engineering and Biotechnology, National Science and Technology Development Agency, Pathum Thani, Thailand; University of Pretoria Faculty of Health Sciences, SOUTH AFRICA

## Abstract

Feline mammary carcinoma (FMC) is the most prevalent reproductive tumor in queens and is characterized by aggressive metastatic progression and short survival. Protein phosphorylation is a crucial process in cell regulation, with dysregulation linked to cancer progression, including human breast cancer. Although phosphoproteins have emerged as diagnostic and predictive markers in human breast cancer, knowledge remains limited on their role in FMC. In this study, the phosphoproteomic profiles of specimens for FMC grades 1 (n = 6), grade 2 (n = 11), grade 3 (n = 14), and normal controls (n = 6) were compared by phosphoprotein enrichment coupled with liquid chromatography–tandem mass spectrometry. Seventeen downregulated phosphoproteins were identified across all FMC grades, many of which have established roles in human breast cancer pathogenesis and prognosis. Serine/threonine–protein phosphatase was identified as a potential growth promoter and therapeutic target, while acid phosphatase, prostate, and ribonuclease L were identified as tumor suppressors. Furthermore, the ABC-type glutathione-S-conjugate transporter was associated with multidrug resistance. Protein kinase AMP-activated noncatalytic subunit gamma 3 was associated with increased breast cancer risk. In this study, it was also found to be associated with Ki-67 expression in FMC (*p* = 0.03). These phosphoproteins interacted with various proteins, immune checkpoint molecules, and chemotherapy drugs associated with mammary cancer in both human and feline species. Furthermore, proteins, such as butyrophilin subfamily 1 member A1, keratin, type I cytoskeletal 10, HECT domain E3 ubiquitin protein ligase 3, nuclear receptor binding SET domain protein 3, and stomatin-like 2, were identified and implicated in cancer progression and prognosis. This study is the first phosphoproteomic investigation of FMC, highlighting the interactions of relevant phosphoproteins with other proteins and chemotherapy drugs associated with both feline and human mammary cancers. The findings provide valuable insights for the identification of diagnostic and prognostic biomarkers and potential therapeutic targets in cats with mammary carcinoma.

## Introduction

Feline mammary carcinoma (FMC) is the most common type of malignant tumor in female cats that arises from the epithelial components of the mammary gland [1]. This malignancy is considered a life-threatening condition because of its aggressive nature and high metastatic potential in both regional and distant organs, presenting significant challenges for clinical management and treatment planning [2,3]. Histopathological grading is crucial in both the diagnosis and prognosis of mammary carcinoma because it indicates the degree of tissue differentiation and disease severity [4,5]. However, many cats are diagnosed with high-grade FMC, complicating treatment options and highlighting the need for improved diagnostic and therapeutic strategies [5,6]. Several factors associated with an increased risk of FMC include older age (10–12 years), specific breeds, such as Persian and Siamese cats, and long-term exposure to exogenous hormones [7,8]. Despite these associations, the underlying mechanisms of mammary carcinoma development remain unclear, requiring further investigation that would improve the current understanding of this disease.

Protein phosphorylation is a common posttranslational modification that mediates signal transduction processes in eukaryotic cells. Phosphorylated proteins, or phosphoproteins, contribute significantly in cellular functions, such as cell growth, transcription, division, movement, and apoptosis. Dysregulation of these proteins has been implicated in the development of several cancers, including human breast cancer [9,10]. Several phosphoproteins have emerged as potential diagnostic biomarkers for human breast cancer [11]. Receptor tyrosine kinases regulate key downstream signaling pathways, such as the mitogen-activated protein kinase, phosphoinositide 3-kinase, and Janus kinase/signal transducer and activator of transcription pathways. These pathways are involved in tumor growth, angiogenesis, and metastasis in breast cancer [12,13]

The emergence of omics technologies and mass spectrometry has revolutionized the rapid and high-throughput profiling of proteins and peptides in biological samples. These technologies are widely used in the veterinary field for biomarker discovery [14–17]. Studies on phosphorylated proteins, known as phosphoproteomics, have proven beneficial in identifying biomarkers for various diseases affecting dogs and cats [18–20]. Phosphoproteomics offers greater sensitivity and specificity for detecting different disease stages than whole proteome analyses [21]. Furthermore, the use of phosphoproteomics in human breast cancer research has enhanced the current understanding of tumorigenesis by uncovering dysregulated signaling pathways in breast cancer cells and their tumor microenvironment [22–25]. Therefore, phosphoproteomics is essential for identifying biomarkers and developing novel targeted therapies for breast cancer [26]. However, phosphoproteomics research in FMC remains limited, highlighting the need for further studies in the veterinary field.

In this study, we conducted phosphoproteomic profiling using phosphoprotein enrichment and nano-liquid chromatography–tandem mass spectrometry (nano-LC-MS/MS) to compare the differences among normal mammary tissues and three FMC tumor grades. This approach enabled the detection of signaling molecules and protein mediators that could serve as biomarkers for FMC. Furthermore, we explored the relationships between the identified candidate biomarkers and other proteins involved in the tumorigenesis and prognosis of FMC, as well as their associations with currently used chemotherapy drugs. Our findings could enhance the pathophysiological understanding of FMC and provide a preliminary foundation for future research on novel biomarkers for diagnosis, prognosis, and targeted therapies.

## Materials and methods

### Animals

This study included 31 female cats with FMC and six controls. All FMC cases were histologically confirmed as mammary carcinoma of any age and neuter status from Prasu Arthorn Veterinary Teaching Hospital, Mahidol University, Thailand between 2021 and 2022. Cases that had previously undergone chemotherapy or had other tumor origins before or at the time of mammary tumor diagnosis were excluded. Specimens of mammary tumors and regional lymph nodes were obtained from patients after surgical resection. For each cat, one tumor tissue was selected for analysis. In cases of multiple tumors, the one with the highest histological grade was chosen for immunohistochemistry staining and phosphoproteomic analysis. Data on signalment and medical history were obtained from medical records. The mean ± standard deviation age of the cats with FMC was 12.2 ± 3.4 years. Fresh normal mammary tissue samples obtained from six cats that underwent necropsy at the Mahidol University Veterinary Diagnostic Center were used as controls. These cats showed no evidence of tumors based on clinical history and necropsy findings. The mean age of the control cats was 7.9 ± 6.4 years (range: 1–12 years). The characteristics of the patients in the FMC and control groups are detailed in S1 Table.

The protocol used in this study was approved by the Mahidol University-Institute Animal Care and Use Committee (MUVS-2021-02-04). Informed consent was obtained from all the owners of the cats enrolled in this study.

For phosphoproteomic analysis, a portion of the fresh mammary tissues (both normal and tumor) was kept at −80°C until used. The remaining mammary tissue and lymph node specimens was preserved in 10% neutral formalin, processed as formalin-fixed paraffin-embedded tissue, and routinely stained with hematoxylin and eosin for histopathological evaluation.

### Histological grading

Histological grading was performed following the novel grading system proposed by Mills et al. [4]. Briefly, the system evaluated nuclear form (score 0, ≤ 5% abnormal nuclear shape; score 1, > 5% abnormal nuclear shape), mitotic count (score 0, ≤ 62 mitoses/10 high-power fields (HPFs); score 1, > 62 mitoses/10 HPFs), and presence of lymphovascular invasion (score 0, absent; score 1, present) using assigned scores. The total score of the three parameters classified the FMC cases into three histological grades: grade 1 (score 0), grade 2 (score 1), and grade 3 (score 2–3).

### Molecular subtype and Ki-67 assessment

To identify the molecular subtype, immunohistochemistry was performed on FMC tissue sections using five antibodies: estrogen receptor (ER), progesterone receptor (PR), feline homologous human epidermal growth factor receptor 2 (*f*HER2), cytokeratin 5/6 (CK5/6), and Ki-67, as previously described [27,28]. Briefly, tissue sections were immersed in 3% (v/v) H_2_O_2_ in methanol for ten min to eliminate endogenous peroxidase. Antigen retrieval was performed using a pressure cooker at 119 °C at 1.5 bar for 15 min in sodium citrate (pH 6.0). The tissue samples were then incubated overnight at 4°C overnight with the following primary antibodies: ER (clone 6F11, dilution 1:100, Thermo Scientific, Waltham, MA), PR (clone 1E2, ready-to-use [RTU], Ventana Medical Systems, Oro Valley, AZ), *f*HER2 (clone CB11, dilution 1:40, Invitrogen, Carlsbad, CA), CK5/6 (clone D5/16, RTU, Dako, Glostrup, Denmark), Ki-67 (clone MIB-1, RTU, Dako, Glostrup, Denmark). Poly-horseradish peroxidase anti-mouse/rabbit IgG (Envision K5007ENV, Dako, Glostrup, Denmark) was used as the secondary antibody and samples were incubated at 37°C for 1 h. Chromogenic reactions were induced by applying 3,3’-diaminobenzidine (DAB) (Envision K5007DAB, Dako, Glostrup, Denmark). All labeled slides were counterstained with Mayer’s hematoxylin. Negative controls were prepared by replacing the primary antibody with homologous non-immune sera. Endometrial epithelium from feline uterine sections served as a positive control for ER and PR. For *f*HER2 and Ki-67, feline mammary carcinoma (FMC) tissues were used as positive controls while normal skin from the mammary tissue served as a positive control for CK5/6. For immunohistochemistry interpretation, ER and PR staining was interpreted according to the Allred score guideline. A tumor was considered positive when a sum score was > 2. *f*HER2 positivity was defined by 2+ or 3 + staining at nuclear membrane and/or cytoplasm. For CK5/6, positive immunoreactivity was defined as > 1% of the tumor cells at cytoplasm and/or cell membrane [27]. Ki-67 index was calculated as a percentage of positive cells by counting positively stained nuclei in a total of 1,000 neoplastic cells and multiplying by ten [29]. In this study, tumor was considered either low or high proliferative based on the mean Ki-67 value.

### Sample preparation for phosphoproteomics

Fresh tumor tissues were ground to powder using liquid nitrogen and then suspended in 0.5% sodium dodecyl sulfate to obtain the protein lysate. The tissue lysates were then centrifuged at 10,000 *g* for 15 min. The supernatant was then transferred into a new tube. Fat and debris were removed from the samples by methanol–chloroform precipitation [30]. The Lowry assay was used to measure the total protein concentration using bovine serum albumin as the standard [31]. The protein concentration of each sample was adjusted to 10 μg/ μL and tested using an immobilized metal ion affinity chromatography (IMAC) phosphoprotein enrichment kit (Pierce phosphoprotein enrichment kit, Thermo Scientific, Rockford, IL). The enriched phosphoprotein was concentrated using a speed vacuum concentrator (Thermo Fisher Scientific, Rockford, IL) and desalted by gel filtration (Thermo Scientific, Rockford, IL). The total phosphoprotein concentration was adjusted to 0.5 µg/µL before undergoing a step of in-solution digestion.

For the in-solution digestion, the enriched tissue samples were dissolved in 10 mM ammonium bicarbonate (NH_4_HCO_3_). Then, 10 mM dithiothreitol in 10 mM NH_4_HCO_3_ was added to react with the samples for 1 h at room temperature to weaken the disulfide bonds of the proteins. Subsequently, alkylation of the sulfhydryl groups was performed using 15 mM iodoacetamide in 10 mM NH_4_HCO_3_ for 1 h at room temperature in the dark. Proteins in each sample were then digested into peptides with sequencing-grade modified trypsin (1:20, Promega Corporation, Madison, WI) for 16 h at 37°C. The tryptic peptides were dried using a speed vacuum concentrator and stored at −20°C until analysis.

### Phosphoproteomic analysis using LC-MS/MS

Total protein samples were prepared for injection into an UltiMate3000 Nano/Capillary LC System (Thermo Scientific, UK) coupled with a ZenoTOF 7,600 mass spectrometer (SCIEX, Framingham, MA, USA). First, the tryptic peptides were resuspended in 0.1% formic acid (FA). Then, peptide digests were enriched on a precolumn packed with PepMap 100 resin (5 µm, 100 A, Thermo Scientific, UK) and separated in a column packed with Acclaim PepMap RSLC C18 resin (2 μm, 100Å, nanoViper, Thermo Scientific, UK). The C18 column was maintained at 60°C using a thermostat-controlled column oven. Solvents A (containing 0.1% FA in water) and B (0.1% FA in 80% acetonitrile) were directed into the analytical column. The peptides were then distinguished using a gradient elution of 5%–55% solvent B at a constant flow rate of 0.30 μL/min for 30 min.

In the ZenoTOF 7,600 system, the precursor ions were dynamically excluded for 12 s after two sets of MS/MS sampling. The MS2 spectra were evaluated in the range of 100–1,800 m/z at a 50-ms accumulation time and with an enabled Zeno trap. The collision energy parameters consisted of a decluttering potential set at 80 V. The time bins were combined using a Zeno trap threshold of 150,000 cps. The cycle time for the Top 60 DDA method was set at 3.0 s. The raw MS/MS spectral data are available on the ProteomeXchange repository under registration numbers JPST0036727 and PXD061475(https://repository.jpostdb.org/preview/48609260067c78082b10a9; Access key: 1810).

MaxQuant 2.4.9.0 was used through the Andromeda search engine to quantify the protein contents of the samples. MS/MS spectra were searched using the UniProtKB/Swiss-Prot *Felis catus* database. The standard settings on MaxQuant for label-free quantitation were a maximum of two missed cleavages, a mass tolerance of 0.6 Das for the main search, trypsin as a digesting enzyme, carbamidomethylation of cysteine as a fixed modification, and oxidation of methionine and acetylation of the protein N-terminus as variable modifications. Peptides with a minimum of seven amino acids and at least one unique peptide were selected for protein identification. At least 2 peptides and at least 1 unique peptide were required for proteins to be included in further data analysis. Reversed search sequences were used to estimate the protein false discovery rate (FDR, set at 1%). Finally, the list of proteins from the *F. catus* proteome was downloaded from UniProt in the FASTA format.

The MaxQuant ProteinGroups.txt file was imported into Perseus version 1.6.6.0 [32]. Contaminants in the data set were removed, and the mass intensities were subjected to log2 transformation. Missing values were imputed in Perseus using a constant value (zero).

Gene ontology enrichment analysis was performed using ShinyGo software (version 0.77) (https://bioinformatics.sdstate.edu/go77/) to classify the biological processes, cellular components, and molecular functions of the identified phosphoproteins [33].

The data of all phosphoproteins were imported into MetaboAnalyst software version 6.0 for data analysis [34]. The expression levels of differential phosphoproteins were illustrated using a heatmap, while the biological processes, cellular components, and molecular functions of the phosphoproteins were determined using UniProtKB/Swiss-Prot (http://www.uniprot.org/).

STITCH 5.0 (http://stitch.embl.de/) was used to predict the functional interaction networks between proteins, small molecules, and chemotherapeutic drugs. This study evaluated differential phosphoproteins significantly identified through the Kruskal–Wallis test; candidate tissue biomarkers previously identified by Paphussaro et al. [35] (i.e., interleukin 18 receptor 1 [IL18R1] and rRNA adenine N[6]-methyltransferase [DIMT1], NOP14 nucleolar protein [NOP14], and sodium channel protein [SCN8A]); immune checkpoint markers (i.e., program cell death 1 [PD-1] and cytotoxic T-lymphocyte protein 4 [CTLA-4]); other associated markers (i.e., estrogen receptor [ESR], progesterone receptor [PGR], epidermal growth factor receptor [EGFR], and Erb-B2 receptor tyrosine kinase 2 [ERBB2]; and chemotherapeutic drugs currently used to treat FMC and human breast cancer (i.e., doxorubicin, cyclophosphamide, 5-fluorouracil, and lapatinib).

### Statistical analysis

Statistical analysis was performed using the R package in MetaboAnalyst (version 6.0) software [6]. The Kruskal–Wallis test, followed by the Mann–Whitney U test, was used to identify differential phosphoproteins among the control and FMC samples. Statistical significance was considered at *p* < 0.01. Differential protein expression levels were compared among molecular subtypes using the Kruskal-Wallis test. Comparisons with Ki-67 expression (low and high) were performed using the Mann-Whitney U test. Statistical significance was defined as *p* < 0.05.

## Results

### Characteristics of feline mammary carcinoma cases

**Fig 1:**
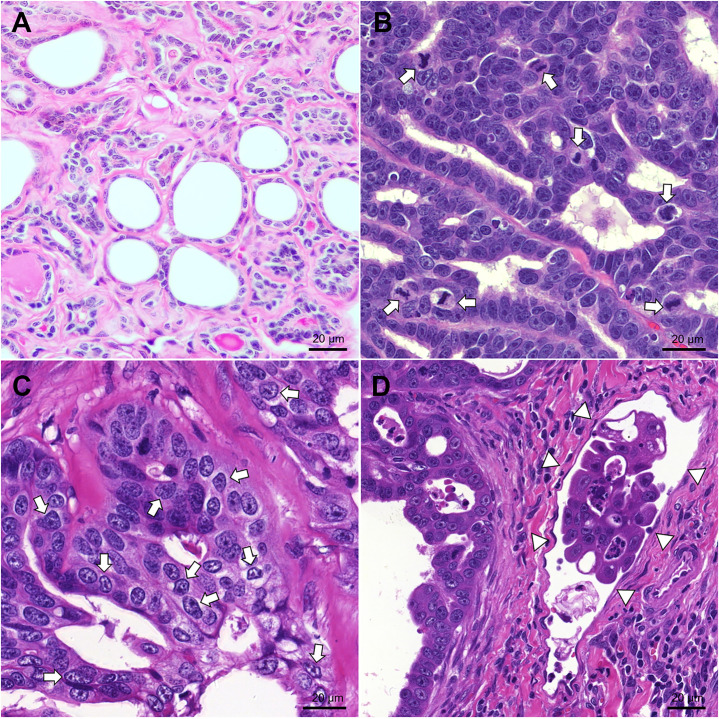
Histopathological findings in normal mammary tissues and various grades of feline mammary carcinoma. **(A)** Normal mammary gland showing lobules lined by a single layer of mammary epithelium, which is supported by a small amount of fibrous connective tissue (hematoxylin and eosin **[H&E]**). Feline mammary carcinoma: **(B)** High mitotic count (arrow), **(C)** >5% abnormal nuclear forms (arrow), and (D) presence of lymphovascular invasion (arrowhead). Scale bar = 20 µm **(H&E)**.

The information of each patient, including signalment, molecular subtype, Ki-67, and metastatic status were detailed in S1 Table. The histopathological findings on the normal and mammary carcinoma tissues are shown in Fig 1. The control group consisted of six normal mammary tissues (Fig 1A). The FMC group included 6 cases (20%) of grade 1, 11 cases (35%) of grade 2, and 14 cases (45%) of grade 3 (Fig 1B**–**1D). In this study, FMC samples included five molecular subtypes: 11 (35.5%) luminal B/ HER2-negative, 5 (16.1%) luminal B/ HER2-positive, 4 (12.9%) HER2-positive, 6 (19.4%) triple-negative basal-like, and 5 (16.1%) triple-negative normal-like (S1 Fig). Mean of Ki-67 expression in FMC tissue were 43.4% ± 15%. Low proliferative tumors were defined when Ki-67 < 43.4%, while high proliferative tumors had Ki-67 ≥ 43.4%. Of 31 cats, nine (29%) showed lymph node metastasis, while lung metastasis was suspected in two cases (6%, S1 Table).

### Phosphoproteins identified by LC-MS/MS

**Fig 2:**
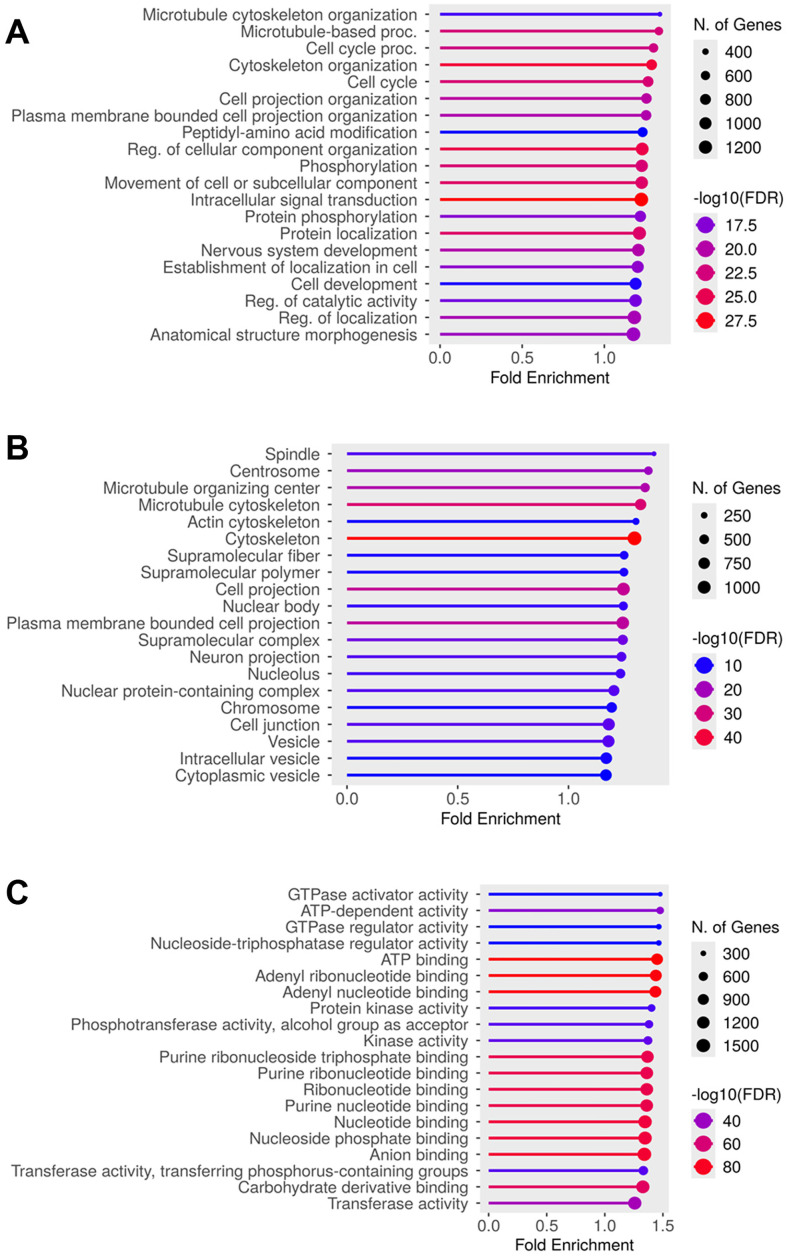
Gene enrichment analysis of all identified phosphoproteins. The phosphoproteins were categorized into (A) biological process, (B) cellular component, and (C) molecular function. The Y-axis shows biological processes, locations, and pathways linked to phosphoproteins, while the X-axis represents fold enrichment (values > 1 indicate overrepresentation). The circle color indicates statistical significance (−log10[FDR]). Purple indicates lower significance, while red indicates higher significance. The circle size represents the number of associated genes.

In total, 11,942 phosphoproteins were identified from the FMC and control samples. According to gene ontology analysis, the major biological processes of phosphoproteins were associated with microtubule cytoskeleton organization, microtubule-based procedure, cell cycle, and cytoskeleton organization (Fig 2A). The phosphoproteins identified were primarily components of the spindle, centrosome, microtubule organization center, microtubule cytoskeleton, and actin cytoskeleton (Fig 2B). The molecular functions of most of the phosphoproteins were related to GTPase activator activity, ATP-dependent activity, GTPase regulator activity, nucleoside-triphosphatase regulator activity, and ATP binding (Fig 2C).

**Fig 3:**
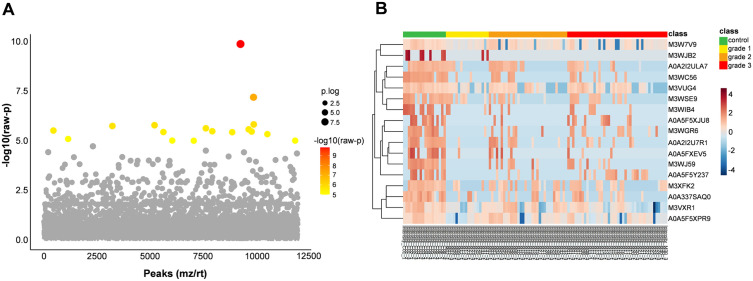
Statistical analysis of the differentially expressed phosphoproteins among the different FMC histological grades. **(A)** The volcano plot illustrates the distribution of significant phosphoproteins identified in the control and FMC groups based on the Kruskal–Wallis test. Of the 11,942 phosphoproteins identified, 17 exhibited significant differences among the normal and FMC grades 1–3 mammary tissues. The x-axis represents the peak of the phosphoprotein identified by their mass-to-charge ratio (m/z) and retention time (rt). The y-axis represents the − log10 of the raw *p* associated with each peak. The colored dots indicate phosphoproteins with a *p* < 0.01, while the gray dot indicates phosphoproteins without statistical significance. **(B)** Heat map showing 17 differentially expressed phosphoproteins among the specimens of different FMC grades and normal mammary tissue. Each column represents a sample categorized into four groups: normal mammary tissue (green, n = 6), grade 1 FMC (yellow, n = 6), grade 2 FMC (orange, n = 11), and grade 3 FMC (red, n = 14). Each row includes phosphoproteins significantly expressed according to the Kruskal–Wallis test (*p* < 0.01). The intensity of color within each cell of the grid represents the level of expression of proteins in the samples. Red denotes protein upregulation, while blue indicates protein downregulation.

The Kruskal–Wallis test was performed and followed by Tukey’s post hoc test to identify the differentially expressed phosphoproteins from FMC samples. The analysis revealed 17 differentially expressed phosphoproteins among the FMC grade and control specimens, including F-box protein 7 (FBXO7), nuclear receptor binding SET domain protein 3 (NSD3), NAD-capped RNA hydrolase NUDT12 (NUDT12), Janus kinase and microtubule interacting protein 2 (JAKMIP2), butyrophilin subfamily 1 member A1 (BTN1A1), Intermediate filament (IF) rod domain-containing protein, ABC-type glutathione-S-conjugate transporter (ABCC3), protein kinase AMP-activated noncatalytic subunit gamma 3 (PRKAG3), keratin, type I cytoskeletal 10 (KRT10), zinc finger BED-type containing 4 (ZBED4), transient receptor potential cation channel subfamily C member 4 associated protein (TRPC4AP), acid phosphatase (ACP3 or ACPP), Serine/threonine-protein phosphatase (PPP1CA), stomatin like 2 (STOML2), HECT domain E3 ubiquitin protein ligase 3 (HECTD3), ribonuclease L r(RNASEL), and NAC-A/B domain-containing protein (Fig 3A, Table 1). Furthermore, the differential phosphoproteins in the FMC group were significantly downregulated compared with those in the control group. However, no significant differences were found in the expression of the differential phosphoproteins among the different FMC grades (Fig 3B, S2 Table). The biological processes, cellular components, and molecular functions of the 17 differential phosphoproteins were annotated using UniProtKB/Swiss-Prot (Table 1).

**Table 1:** Differentially expressed phosphoproteins of feline mammary carcinoma based on statistical analysis and UniProtKB/Swiss-Prot.

Protein ID	Protein name	Peptide sequence	−log 10(p)	FDR	Biological process	Cellular component	Molecular function
M3WC56	F-box protein 7 (FBXO7)	MARRPGG	9.86	1.64 × 10^ − 6^	lymphocyte differentiation, negative regulation of the G1/S transition of the mitotic cell cycle, negative regulation of lymphocyte differentiation, negative regulation of the oxidative stress-induced neuron intrinsic apoptotic signaling pathway, positive regulation of the autophagy of mitochondrion, positive regulation of mitophagy, proteasome-mediated ubiquitin-dependent protein catabolic process, protein K48-linked ubiquitination, protein targeting to mitochondrion, regulation of locomotion, regulation of neuron projection development, regulation of protein stability	classical Lewy body, cytosol, glial cytoplasmic inclusion, Lewy body core, Lewy body corona, Lewy neurite, mitochondrion, nucleoplasm, SCF ubiquitin ligase complex	protein heterodimerization activity, protein kinase binding, ubiquitin binding, ubiquitin protein ligase binding, ubiquitin-like ligase-substrate adaptor activity
M3WJ59	Nuclear receptor binding SET domain protein 3 (NSD3)	MDFSFSF	7.18	3.91 × 10^ − 4^	positive regulation of DNA-templated transcription, regulation of DNA-templated transcription	chromatin, nucleus	histone H3K27 dimethyl transferase activity, histone H3K27 trimethyl transferase activity, histone H3K36 methyltransferase activity, histone H3K4 dimethyl transferase activity, metal ion binding, transcription regulator activator activity
M3WJB2	NAD-capped RNA hydrolase NUDT12 (NUDT12)	MKMKGFF	5.82	3.73 × 10^ − 3^	mRNA methylguanosine-cap recapping, NAD catabolic process, NAD-cap recapping, NADH metabolic process, NADP catabolic process	nucleus, peroxisome	metal ion binding, NAD+ diphosphatase activity, NADH pyrophosphatase activity, phosphodiesterase recapping endonuclease activity
A0A5F5XEV5	Janus kinase and microtubule-interacting protein 2 (JAKMIP2)	ERMELLQ	5.78	3.73 × 10^ − 3^	n/a	n/a	kinase binding, microtubule binding
A0A337SAQ0	Butyrophilin subfamily 1 member A1 (BTN1A1)	MDFPALW	5.74	3.73 × 10^ − 3^	regulation of cytokine production, T cell receptor signaling pathway	external side of the plasma membrane	signaling receptor binding
M3VUG4	IF rod domain-containing protein	MTQRSSV	5.62	3.73 × 10^ − 3^	intermediate filament organization and keratinization	keratin filament	structural constituents of the skin epidermis
M3WGR6	ABC-type glutathione-S-conjugate transporter (ABCC3)	FQNSLLA	5.57	3.73 × 10^ − 3^	leukotriene transport, transmembrane transport, xenobiotic transmembrane transport	basolateral plasma membrane, membrane	ABC-type glutathione S-conjugate transporter activity, ABC-type xenobiotic transporter activity, ATP binding, ATP hydrolysis activity, ATPase-coupled transmembrane transporter activity, glucuronoside transmembrane transporter activity, icosanoid transmembrane transporter activity
A0A2I2U7R1	Protein kinase AMP-activated noncatalytic subunit gamma 3 (PRKAG3)	MWCPACL	5.51	3.73 × 10^ − 3^	cellular response to nutrient levels, fatty acid biosynthetic process, glycogen biosynthetic process, glycolytic process, response to muscle activity involved in the regulation of muscle adaptation	cytoplasm, nucleotide-activated protein kinase complex, nucleus	AMP binding, AMP-activated protein kinase activity, protein kinase binding, protein kinase regulator activity
M3VXR1	Keratin, type I cytoskeletal 10 (KRT10)	MSVRYSS	5.47	3.73 × 10^ − 3^	epidermis development, epithelial cell differentiation, intermediate filament organization, keratinocyte differentiation, protein heterotetramerization	cell surface, cornified envelope, cytoplasm, cytoskeleton, extracellular region, keratin filament	protein heterodimerization activity and the structural constituents of the skin epidermis
M3WIB4	Zinc finger BED-type containing 4 (ZBED4)	MENNQEPR	5.46	3.73 × 10^ − 3^	n/a	cytoplasm, nucleoplasm	DNA-binding transcription activator activity, RNA polymerase II-specific, identical protein binding, metal ion binding, protein dimerization activity, RNA binding, RNA polymerase II transcription regulatory region sequence-specific DNA binding
A0A5F5XJU8	Transient receptor potential cation channel subfamily C member 4 associated protein (TRPC4AP)	MAAAPAA	5.43	3.73 × 10^ − 3^	ubiquitin-dependent protein catabolic process	Cul4A-RING E3 ubiquitin ligase complex	
M3W7V9	acid phosphatase or acid phosphatase, prostrate (ACP3 or ACPP)	MSAVPLP	5.43	3.73 × 10^ − 3^	adenosine metabolic process, dephosphorylation, lysosome organization, nucleotide metabolic process, positive regulation of the adenosine receptor signaling pathway, purine nucleobase metabolic process, regulation of the sensory perception of pain, thiamine metabolic process	extracellular space, filopodium, lysosome, plasma membrane, vesicle membrane	5′-nucleotidase activity, acid phosphatase activity, lysophosphatidic acid phosphatase activity, phosphatase activity, protein homodimerization activity, thiamine phosphate phosphatase activity
M3WSE9	Serine/threonine-protein phosphatase (PPP1CA)	MSDSEKL	5.33	4.31 × 10^ − 3^	branching morphogenesis of an epithelial tube, cell division, circadian regulation of gene expression, dephosphorylation, entrainment of the circadian clock by the photoperiod, glycogen metabolic process, lung development, positive regulation of the extrinsic apoptotic signaling pathway in the absence of ligand	adherent junction, chromosome, telomeric region, cytoplasm, cytosol, nucleoplasm, nucleus, plasma membrane, PTW/PP1 phosphatase complex	cadherin binding involved in cell-cell adhesion, myosin phosphatase activity, and protein serine/threonine phosphatase activity
A0A2I2ULA7	Stomatin like 2 (STOML2)	MLARAAR	5.09	7 × 10^ − 3^	n/a	membrane, mitochondrion	n/a
A0A5F5XPR9	HECT domain E3 ubiquitin protein ligase 3 (HECTD3)	MAGPGPG	5.00	7.09 × 10^ − 3^	n/a	n/a	ubiquitin-protein transferase activity
M3XFK2	Ribonuclease L (RNASEL)	MESKNHN	5.00	7.09 × 10^ − 3^	defense response to virus, fat cell differentiation, mRNA processing, negative regulation of viral genome replication, positive regulation of glucose import, positive regulation of transcription by RNA polymerase II, regulation of mRNA stability, RNA processing	n/a	ATP binding, protein kinase activity, ribonucleoprotein complex binding, RNA binding, RNA nuclease activity
A0A5F5Y237	NAC-A/B domain-containing protein	MGSLSAA	5.00	7.09 × 10^ − 3^	protein targeting the membrane	cytoplasm, nascent polypeptide-associated complex	unfolded protein binding

FDR: false discovery rate.

**Fig 4:**
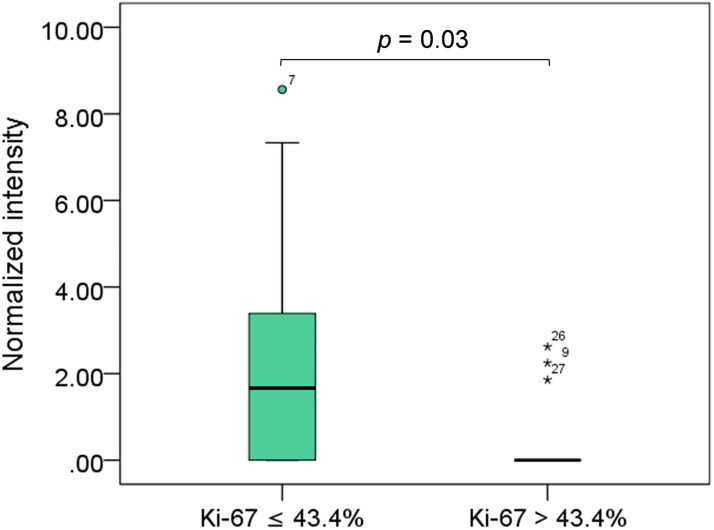
Box-plots showed a significant association between expression level of protein kinase AMP-activated noncatalytic subunit gamma 3 (PRKAG3) and Ki-67 expression. Low proliferation was defined as Ki-67 ≤ 43.4%, and high proliferation as Ki-67 > 43.4%. For PRKAG3 expression, the median [interquartile range] for the low proliferation group was 1.66 [0.00, 4.38], and for the high proliferation group was 0.00 [0.00, 0.46]. A statistical difference was calculated using the Mann Whitney test.

In this study, there was no significantly among 17 differential phosphoproteins and five molecular subtypes of FMC (S3 Table). However, PRKAG3 were significantly associated with Ki-67 expression. Low proliferative tumors (Ki-67 ≤ 43.4%) had higher expression of PRKAG3 compared to high proliferative tumor (Ki-67 > 43.4%, *p* = 0.03, Fig 4).

**Fig 5:**
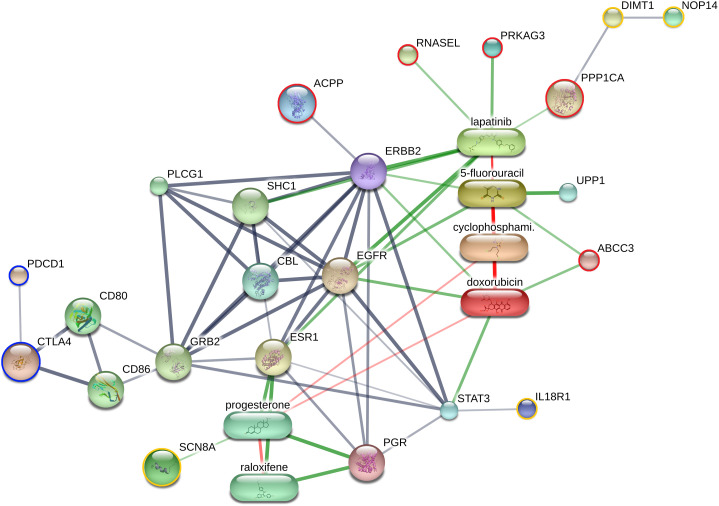
Interaction between the identified phosphoproteins, immune checkpoints,associated markers, and chemotherapeutic drugs cyclophosphamide, doxorubicin, 5-fluorouracil, and lapatinib in protein–protein and protein–drug interaction networks. The red circle represents the differentially expressed phosphoproteins, while the yellow circle indicates the candidate proteins identified in a previous study [35]. The blue circle represents the immune checkpoint markers associated to feline mammary carcinoma [36,37]. The strength of the functional interactions is indicated by edge confidence scores. The thickest lines represented the highest confidence score (>0.90). The predicted functional partners included the Cbl protooncogene, E3 ubiquitin protein ligase (CBL), growth factor receptor-bound protein 2 (GRB2), phospholipase C, gamma 1 (PLCG1), SHC transforming protein 1 (SHC1), signal transducer and activator of transcription 3 (STAT3), T-lymphocyte activation antigen CD80 (CD80), T-lymphocyte activation antigen CD86 precursor (CD86), transforming growth factor alpha, and uridine phosphorylase 1 (UPP1). Abbreviations: ATP-binding cassette, subfamily C, member 3 (ABCC3); Acid phosphatase, prostate (ACPP); cytotoxic T-lymphocyte protein 4 (CTLA4); rRNA adenine N(6)-methyltransferase (DIMT1); epidermal growth factor receptor (EGFR); receptor tyrosine-protein kinase erbB 2 precursor (ERBB2); estrogen receptor (ESR); gasdermin C (GSDMC); interleukin-18 receptor 1 (IL18R1); NOP14 nucleolar protein (NOP14); programmed cell death 1 (PDCD1); progesterone receptor (PGR); protein phosphatase 1, catalytic subunit, alpha isozyme (PPP1CA); protein kinase, AMP-activated, gamma 3 noncatalytic subunit (PRKAG3); ribonuclease L (RNASEL); sodium channel protein (SCN8A).

Among the 17 differential phosphoproteins, ABCC3, ACPP, PPP1CA, PRKAG3, and RNASEL were associated with FMC-related markers (i.e., EGFR, ERBB2, ESR, and PGR), and immune checkpoint markers (i.e., cytotoxic T-lymphocyte protein 4 [CTLA4], and programmed cell death 1 [PD-1]. The protein–drug network revealed that these phosphoproteins were associated with several chemotherapeutic agents, including doxorubicin, 5-fluorouracil, and lapatinib. Furthermore, the network showed interactions between five differential phosphoproteins, chemotherapeutic drugs, and previously identified FMC tissue biomarkers using proteomics, including DIMT1, IL18R1, NOP14, and SCN8A [35]. However, the other 12 differential phosphoproteins were not identified in the protein–protein and protein–drug networks (Fig 5).

## Discussion

This study compared the phosphoproteomic profiles between different FMC grades and normal mammary tissue using phosphoprotein enrichment followed by LC-MS/MS. FMC grading was performed to assess variations in phosphoprotein expression across tumor progression stages. IMAC was used as the enrichment technique because of its commercial availability and simple preparation process [38,39]. The comparative analysis between normal and tumor tissues with different FMC grades would provide valuable insights into the molecular changes associated with tumor progression and potential biomarkers for disease diagnosis and prognosis. Seventeen differential phosphoproteins were downregulated across all FMC grades compared with the controls (Table 1). Among these, ABCC3, ACPP, PPP1CA, PRKAG3, and RNASEL exhibited interactions with several proteins, immune checkpoint markers, and chemotherapeutic drugs administered in both human breast cancer and FMC (Fig 5). Moreover, PRKAG3 was associated with Ki-67 expression in FMC (Fig 4). While, other phosphoproteins (e.g., BTN1A1, KRT10, HECTD3, NSD3, and STOML2) were associated with human breast cancer prognosis. However, our study did not find differences between differential phosphoproteins and molecular subtypes of FMC (S3 Table).

ABCC3 is a transmembrane transporter involved in various physiological processes [40,41]. In human breast cancer, ABCC3 is overexpressed in cancerous tissue, particularly in patients with human epidermal growth factor receptor 2 (HER2)-positive breast cancer [42]. Moreover, it is also associated with multidrug resistance and poor prognosis. Downregulation of ABCC3 increases drug retention and sensitivity to doxorubicin and 5-fluorouracil in breast and colorectal cancers [43,44]. However, in the present study, ABCC3 expression was low among all FMCs compared with healthy mammary tissue and did not differ among molecular subtypes. This finding contrasts with its typical role in human cancers, highlighting the need for further investigation into the role of ABCC3 and underlying mechanisms in drug resistance in feline cancers.

ACPP is a lysosomal enzyme involved in the metabolism of phosphates and the regulation of cellular signaling pathways [45]. In the current study, ACPP expression was lower in FMC than in normal mammary tissues and was potentially associated with ERBB2 expression. This finding aligned with findings in human malignancies, where ACPP is highly expressed in breast cyst fluid and normal mammary tissue but is absent in breast cancer [46]. A previous study proposed that ACPP inhibits mammary epithelial growth via TGF-β activation [47], but the mechanism remains unclear and requires further investigation. In prostate cancer, ACPP functions as a tumor suppressor by dephosphorylating ERBB2. [48]. Therefore, the low expression of ACPP in FMC may induce the growth of mammary tumors in patients.

PPP1CA is a member of the phosphoprotein phosphatase catalytic subunit family involved in human breast cancer development and progression. Xie et al. [49] reported higher PPP1CA expression in breast cancer tissue than in the surrounding normal mammary tissue. Overexpression of *PPP1CA* is correlated with poor prognosis, older age, lymph node metastasis, and HER2-positive status. A previous study suggested that PPP1CA may promote tumor proliferation by disrupting cell cycle regulation. [50]. Thus, blocking this marker could potentially suppress breast cancer growth and metastasis [49]. However, our study found reduced PPP1CA expression in FMC tissues, with no variation across tumor grades or molecular subtypes. Therefore, further studies are needed to validate this finding.

PRKAG3, a regulatory subunit of the AMP-activated protein kinase (AMPK) complex, has been associated with breast cancer risk in several studies. Chen et al. [51] demonstrated the predictive value of PRKAG3 in patients with triple-negative breast cancer with an accuracy of 91.7%. Another study associated *PRKAG3* with the mammalian target of rapamycin (mTOR) pathway and breast cancer risk, particularly among patients with estrogen receptor-negative breast cancer [52]. In our study, PRKAG3 expression was downregulated in FMC tissues compared with that in controls. Because AMPK functions as a tumor suppressor by inhibiting the mTOR pathway [53], we hypothesized that the decreased PRKAG3 activity in mammary carcinoma may decrease the inhibitory effect of AMPK, leading to tumor growth. Moreover, our study observed a significant association between low PRKAG3 expression and high Ki-67 expression (> 43.4%). As the Ki-67 index, proliferative marker, has been used as a prognostic marker for FMC, [28,54] cats with low PRKAG3 may indicate a worse prognosis. However, this requires further investigation for confirmation.

RNASEL is an endoribonuclease that functions as a tumor suppressor [55]. *RNASEL* mutations can reduce the enzymatic activity and impair the proapoptotic function of its protein, contributing to the development of human prostate cancer [56]. However, the role of RNASEL in human breast cancer remains controversial [57,58]. Our study found decreased RNASEL expression in FMC cats, indicating that its reduced tumor-suppressing activity contributes to tumor growth and metastasis. Therefore, further investigation into the role of RNASEL in mammary tumors is warranted.

In this study, we observed indirect association between differentially expressed phosphoproteins in FMC tissue and immune checkpoint molecules, specifically CTLA-4 and PD-1 (**Fig 5**). Immune checkpoint inhibitors have become a current novel treatment strategy for human breast cancer and are gaining interested in FMC [59]. They function by blocking inhibitory pathways that regulate the immune response, which in turn permits immune cells to activate and effectively eliminate cancer cells. Important immune checkpoint molecules in FMC include CTLA-4, PD-1, and V-domain immunoglobulin suppressor of T cell activation (VISTA) [36,37,60]

CTLA-4, a key transmembrane receptor expressed on regulatory T cells (Tregs), was found to be elevated in the serum of cats with FMC [37], PPP1CA is known to impair the function of Tregs by inactivating FOXP3 [61]. Therefore, the reduced expression of PPP1CA in FMC may lead to diminished inhibition of Tregs and support or enhance CTLA-4’s suppressive signaling, contributing to an immune-suppressive environment. Consequently, the use of an immune checkpoint inhibitor targeting CTLA-4 may be beneficial for FMC patients.

PD-1 is expressed on lymphoid and myeloid cells, including T cell, and its expression was found to be significantly higher in cats with HER2-positive FMC [36]. PRKAG3, as part of the AMPK complex, plays a role in immune cell function and has been implicated in promoting CD8 + T cell exhaustion, contributing to tumor immune escape [62]. Although no direct association between PRKAG3 and PD-1 has been established, the downregulation of PRKAG3 observed in this study may lead to reduced CD8 + T cell exhaustion. This could potentially enhance anti-tumor immune responses and synergize with anti-PD-1 therapy.

In human breast cancer, high expression levels of KRT10, HECTD3, NSD3, and STOML2 were associated with unfavorable outcomes, whereas high BTN1A1 expression was linked to a better prognosis. Furthermore, KRT10 may be a tumor differentiation marker, with 16% of patients with breast cancer testing positive for it. Moreover, gene expression analysis indicated that high KRT10 levels were associated with shorter relapse-free and overall survival times in breast cancer [63]. HECTD3 is expressed in various human organs, including the mammary glands. A previous study found that HECTD3 promoted breast cancer cell survival and may be a potential diagnostic and prognostic biomarker for breast cancer. Specifically, HECTD3 exhibits an approximately twofold higher expression in breast cancer cell lines than normal mammary gland tissues [64]. HECTD3 overexpression is also associated with cisplatin resistance, indicating that HECTD3 inhibitor therapy may benefit patients exhibiting chemoresistance [65]. NSD3 plays a crucial role in the maintenance of genome integrity, and *NSD3* is an important oncogene in breast cancer [66–68]. Jeong et al. [69] highlighted the role of NSD3 in the epigenetic regulation of breast cancer by promoting epithelial–mesenchymal transition (EMT), leading to tumor invasion and metastasis. STOML2, a mitochondrial protein, was previously reported by Cao et al. [70] to be overexpressed in breast cancer compared to normal mammary tissues. High STOML2 levels are associated with breast cancer progression and poor prognosis. A recent study found that STOML2 promotes the viability and motility of breast cancer cells in vitro by mediating FOXO3a expression and the extracellular signal-regulated kinase (ERK) pathway, indicating its potential as a therapeutic target for breast cancer [71]. As a member of the butyrophilin family, BTN1A1 contributes to immune regulation by acting as an immune checkpoint to inhibit T-cell activation [72]. It was previously proposed as a biomarker for breast cancer and a potential therapeutic target. High BTN1A1 expression was correlated with better overall survival in breast cancer [73].

No direct association was observed between TRPC4AP and NUDT12 and mammary tumors. Nevertheless, other phosphoproteins in the same family were associated with human breast cancer. Similar to TRPC4AP, transient receptor potential vanilloid 4 belongs to the transient receptor potential superfamily. It may play a role in cancer extravasation, which causes breast cancer metastasis, and is associated with an aggressive subtype [74]. NUDT1, NUDT2, NUDT5, and NUDT16 were overexpressed in breast cancer tissue. High levels of these proteins were linked to shorter overall survival in hormonal receptor-positive patients. Moreover, NUDT5 also initiated EMT and contributed to breast cancer metastasis [75]

The other phosphoproteins, including FBXO7 and JAKMIP2, were not associated with mammary tumors in humans or animals. Nonetheless, they were linked to other types of tumors. FBXO7 acts as a tumor suppressor by inhibiting serine synthesis. It was downregulated in patients with hepatocellular carcinoma [76]. JAKMIP2 promotes the proliferation of colorectal cancer. Upregulation of this protein is considered a poor prognostic indicator [77]. Currently, no information is available on the role of IF rod domain-containing protein, NAC-A/B domain-containing protein, and ZBED4 in human and animal cancers.

Gene ontology analysis provided valuable insights into the pathways associated with the phosphoproteins identified in FMC tissues (Fig 2). The results indicated an association between phosphoproteins and microtubule cytoskeleton organization or cell cycle regulation, both of which are crucial biological processes for maintaining cell structure and facilitating cell division and intracellular transport, which are frequently dysregulated in cancer cells [48,78,79]. Furthermore, several of the molecular function pathways identified were involved in energy metabolism. These pathways are vital for cell proliferation and survival [80]. Understanding these gene enrichment pathways provides important insights into tumor biology and highlights potential therapeutic targets for FMC.

This study has some limitations. First, one of the primary challenges in phosphoproteomic studies is the relatively low concentrations of phosphoproteins obtained, necessitating the use of enrichment methods to improve detection. However, this approach may not have eliminated the risk of overlooking important phosphoproteins [81].The second limitation is the use of mammary tissue samples, an invasive method. However, these sample provide the highest concentration of potential biomarkers and can be a foundation for future studies in non-invasive specimens, such as blood, urine, and other biofluids [82]. Phosphoproteomic analyses also require specialized expertise in sample preparation and data interpretation. Furthermore, the small sample size and variations in physiological factors (e.g., age, breed, and reproductive status) may have affected the statistical significance of the results, primarily in terms of sample availability and cost constraints. Moreover, the lack of follow-up data in this study limited the ability to perform a comprehensive prognostic evaluation of differential phosphoproteins.

Despite these limitations, this study is the first to investigate phosphoproteomics in feline mammary cancer, offering insights into its similarities and differences with human breast cancer. Future studies with larger sample sizes are needed to validate and expand upon the study findings.

## Conclusion

This study identified tissue phosphoproteins uniquely expressed in feline mammary carcinoma using an IMAC enrichment method followed by LC-MS/MS analysis. We found that ABCC3, ACPP, PPP1CA, PRKAG3, and RNASEL were involved in the tumorigenesis of FMC and exhibited interactions with proteins and chemotherapeutic drugs relevant to both human breast cancer and FMC. In contrast, BTN1A1, KRT10, HECTD3, NSD3, and STOML2 were linked to disease progression and prognosis. This research provides valuable insights into the tissue phosphoproteins involved in FMC, which could serve as biomarkers for developing diagnostic, prognostic, and therapeutic targets in FMC. Future studies should validate the present results and further explore the roles of the phosphoproteins in FMC identified in this study.

## Supporting information

S1 Fig Identification of molecular subtypes in FMC based on ER, PR, HER2, CK5/6, and Ki-67 expression. Luminal B/HER2-negative (LB/ HER2 − , n = 11) was defined as ER or PR + , *f*HER2 − , Ki-67 ≥ 14%. Luminal B/ HER2-positive (LB/ HER2 + , n = 5) was defined as ER or PR + , HER2 + , Ki-67 ≥ 14%. HER2-positive (HER2 + , n = 4) was defined as ER − , PR− and HER2 + . Triple negative/ basal-like (TN-BL, n = 6) was define as ER − , PR − , fHER2 − , and CK5/6 + . Triple negative/ normal-like (TB-NL, n = 5) was defined as ER − , PR − , HER2 − , and CK5/6 − . Abbreviations: ER, estrogen receptor; PR, progesterone receptor; HER2, human epidermal growth factor receptor 2; CK5/6, cytokeratin 5/6.
(TIF)

S1 Table Characteristics of the patients with feline mammary carcinoma and normal mammary tissues. (PDF)

S2 Table Comparison of the differentially expressed phosphoproteins among normal mammary tissue and different histological grades of mammary carcinoma by liquid chromatography–tandem mass spectrometry. (PDF)

S3 Table Comparison of differentially expressed phosphoproteins among molecular subtypes of feline mammary carcinoma. (DOCX)
