# Supplementary figures and images for: Phosphoproteomic profiling of feline mammary carcinoma: Insights into tumor grading and potential therapeutic targets
Source: PLoS One. 2025 Aug 21;20(8):e0330520. doi: 10.1371/journal.pone.0330520 (PMC12370146; doi:10.1371/journal.pone.0330520)

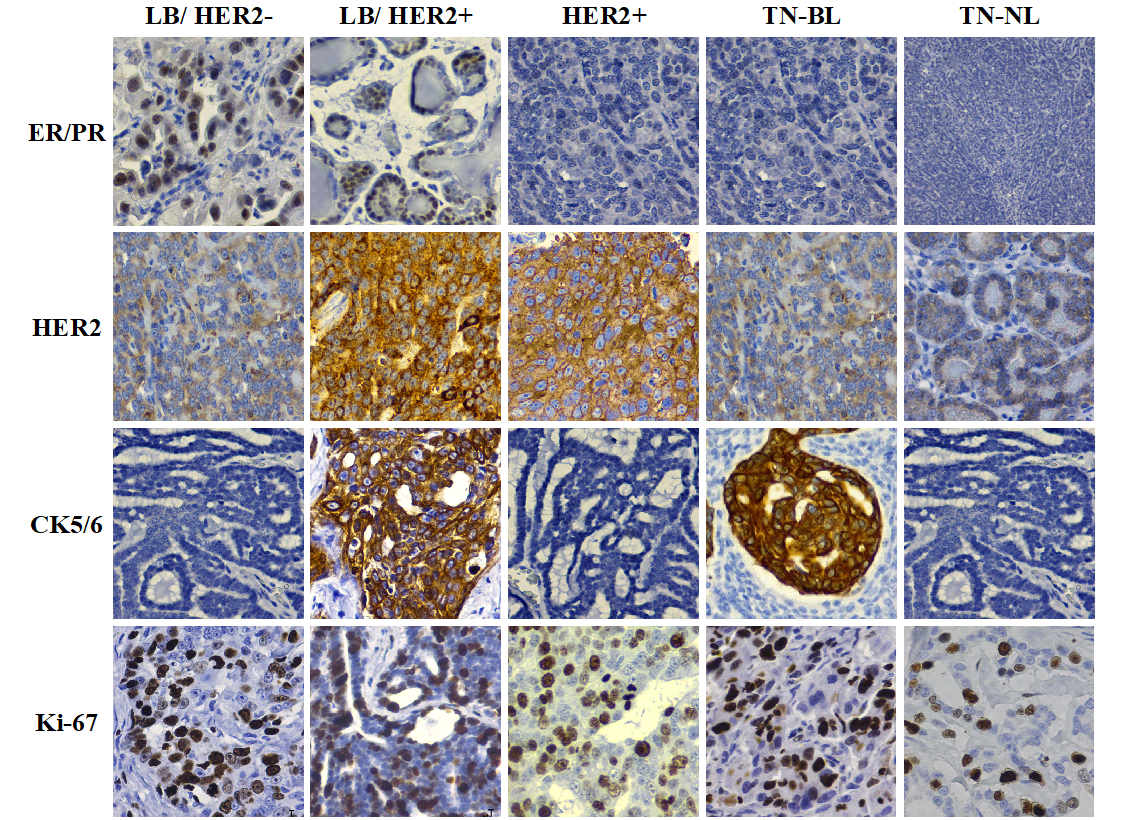

Supplement: S1 Fig — Luminal B/HER2-negative (LB/ HER2 − , n = 11) was defined as ER or PR + , fHER2 − , Ki-67 ≥ 14%. Luminal B/ HER2-positive (LB/ HER2 + , n = 5) was defined as ER or PR + , HER2 + , Ki-67 ≥ 14%. HER2-positive (HER2 + , n = 4) was defined as ER − , PR− and HER2 + . Triple negative/ basal-like (TN-BL, n = 6) was define as ER − , PR − , fHER2 − , and CK5/6 + . Triple negative/ normal-like (TB-NL, n = 5) was defined as ER − , PR − , HER2 − , and CK5/6 − . Abbreviations: ER, estrogen receptor; PR, progesterone receptor; HER2, human epidermal growth factor receptor 2; CK5/6, cytokeratin 5/6. (TIF) [file pone.0330520.s001.tif]
