# Supplementary material for: Phosphoproteomic profiling of feline mammary carcinoma: Insights into tumor grading and potential therapeutic targets
Source: PLoS One. 2025 Aug 21;20(8):e0330520. doi: 10.1371/journal.pone.0330520 (PMC12370146; doi:10.1371/journal.pone.0330520)
Supplement: S1 Table — (PDF) [file pone.0330520.s002.pdf]

S1 Table. Characteristics of the patients with feline mammary carcinoma and normal mammary tissues.

Sample No.	Histological grade	Age (years)	Breed	Neuter status	Molecular subtype	Ki-67	Metastasis status	
							Lymph node	Lung
1	normal mammary tissue	21	Domestic shorthair	Intact	-	-	-	-
2	normal mammary tissue	10	Domestic shorthair	OVH	-	-	-	-
3	normal mammary tissue	7	Domestic shorthair	OVH	-	-	-	-
4	normal mammary tissue	1	Domestic shorthair	OVH	-	-	-	-
5	normal mammary tissue	4	Domestic shorthair	Intact	-	-	-	-
6	normal mammary tissue	4	American shorthair	Intact	-	-	-	-
7	Grade 1 FMC	12	American shorthair	OVH	LB/HER2+	35%	N/A	Absent
8	Grade 1 FMC	18	Domestic shorthair	OVH	TN-BL	31%	N/A	Absent
9	Grade 1 FMC	10	Domestic shorthair	Intact	TN-NL	15%	Absent	Absent
10	Grade 1 FMC	12	Persian	OVH	TN-BL	48%	Absent	Absent
11	Grade 1 FMC	17	Domestic shorthair	OVH	LB/HER2−	23%	N/A	Absent
12	Grade 1 FMC	10	Domestic shorthair	OVH	TN-BL	55%	N/A	Absent

Sample No.	Histological grade	Age (years)	Breed	Neuter status	Molecular subtype	Ki-67	Metastasis status	
							Lymph node	Lung
13	Grade 2 FMC	13	Domestic shorthair	Intact	HER2+	41%	Present	Absent
14	Grade 2 FMC	12	Persian	OVH	LB/HER2+	39%	Present	Absent
15	Grade 2 FMC	10	Persian	OVH	LB/HER2−	45%	N/A	Absent
16	Grade 2 FMC	8	Domestic shorthair	OVH	LB/HER2−	43%	N/A	Absent
17	Grade 2 FMC	12	Domestic shorthair	OVH	LB/HER2−	65%	Absent	Absent
18	Grade 2 FMC	11	Persian	OVH	LB/HER2−	35%	Absent	Absent
19	Grade 2 FMC	10	Domestic shorthair	OVH	LB/HER2−	35%	Present	Absent
20	Grade 2 FMC	12	Domestic shorthair	Intact	TN-BL	30%	Absent	Present
21	Grade 2 FMC	13	Persian	Intact	HER2+	38%	Present	Absent
22	Grade 2 FMC	15	Domestic shorthair	OVH	HER2+	19%	N/A	Absent
23	Grade 2 FMC	10	Domestic shorthair	OVH	LB/HER2−	47%	Present	Absent
24	Grade 3 FMC	6	Domestic shorthair	OVH	TN-NL	32%	Present	Absent
25	Grade 3 FMC	8	Domestic shorthair	OVH	LB/HER2−	53%	Present	Absent
26	Grade 3 FMC	12	Domestic shorthair	Intact	TN-NL	27%	N/A	Absent
27	Grade 3 FMC	11	Persian	Intact	TN-BL	31%	Present	Present
28	Grade 3 FMC	14	Domestic shorthair	OVH	TN-BL	39%	Present	Absent
29	Grade 3 FMC	13	Persian	Intact	TN-NL	54%	Present	Present
30	Grade 3 FMC	20	Domestic shorthair	Intact	LB/HER2−	37%	Absent	Absent

Sample No.	Histological grade	Age (years)	Breed	Neuter status	Molecular subtype	Ki-67	Metastasis status	
							Lymph node	Lung
31	Grade 3 FMC	14	Domestic shorthair	OVH	LB/HER2−	53%	Present	Absent
32	Grade 3 FMC	15	Persian	OVH	HER2+	51%	N/A	Absent
33	Grade 3 FMC	13	Domestic shorthair	OVH	LB/HER2+	67%	N/A	Absent
34	Grade 3 FMC	11	Siamese	OVH	LB/HER2+	72%	Present	N/A
35	Grade 3 FMC	11	Domestic shorthair	OVH	TN-NL	73%	Present	Absent
36	Grade 3 FMC	6	Domestic shorthair	OVH	LB/HER2−	67%	Present	Absent
37	Grade 3 FMC	20	Domestic shorthair	OVH	LB/HER2+	48%	Present	Absent

Abbreviations: FMC, feline mammary carcinoma; HER2+, HER2-positive; LB/HER2−, luminal B HER2-negative; LB/HER2+, luminal B HER2-positve; OVH, Ovariohysterectomy; TN-BL, triple negative basal-like; TN-NL, triple negative normal-like.
