# Supplementary material for: Phosphoproteomic profiling of feline mammary carcinoma: Insights into tumor grading and potential therapeutic targets
Source: PLoS One. 2025 Aug 21;20(8):e0330520. doi: 10.1371/journal.pone.0330520 (PMC12370146; doi:10.1371/journal.pone.0330520)
Supplement: S2 Table — (PDF) [file pone.0330520.s003.pdf]

S2 Table. Comparison of the differentially expressed phosphoproteins among normal mammary tissue and different histological grades of mammary carcinoma by liquid chromatography–tandem mass spectrometry.

Protein name (peptide sequence)	Gene name	Control		Grade 1 FMC		Grade 2 FMC		Grade 3 FMC		-log 10c(*p*)	FDR
		Median	IQR	Median	IQR	Median	IQR	Median	IQR		
F-box protein 7 (MARRPGG)	FBXO7	8.51^{a}	0.41	0^{b}	0	0^{b}	0	0^{b}	0	9.86	1.64×10^{-6}
Nuclear receptor binding SET domain protein 3 (MDFSFSF)	NSD3	7.73^{a}	1.35	0^{b}	0	0^{b}	0	0^{b}	0	7.18	3.91×10^{-4}
NAD-capped RNA hydrolase NUDT12 (MKMKGFF)	NUDT12	0^{a}	8.74	0^{b}	0	0^{b}	0	0^{b}	0	5.82	3.73×10^{-3}
Janus kinase and microtubule interacting protein 2 (ERMELLQ)	JAKMIP2	6.93^{a}	8.17	0^{b}	0	0^{b}	0	0^{b}	0	5.78	3.73×10^{-3}
Butyrophilin subfamily 1 member A1 (MDFPALW)	n/a	7.34^{a}	1.52	0^{b}	0	0^{b}	5.65	0^{b}	5.39	5.74	3.73×10^{-3}
IF rod domain-containing protein (MTQRSSV)	LOC101097497	9.31^{a}	0.77	6.97^{b}	8.13	8.30^{b}	8.99	7.52^{b}	8.96	5.62	3.73×10^{-3}

Protein name (peptide sequence)	Gene name	Control		Grade 1 FMC		Grade 2 FMC		Grade 3 FMC		-log 10c(p)	FDR
		Median	IQR	Median	IQR	Median	IQR	Median	IQR		
ABC-type glutathione-S-conjugate transporter (FQNSLLA)	ABCC3	7.26[a]	7.57	0[b]	0	0[b]	0	0[b]	6.07	5.57	3.73×10^{-3}
Protein kinase AMP-activated non-catalytic subunit gamma 3 (MWCPACL)	PRKAG3	7.29[a]	1.26	0[b]	0	0[b]	6.74	0[b]	0	5.51	3.73×10^{-3}
Keratin, type I cytoskeletal 10 (MSVRYSS)	KRT10	14.42[a]	1.14	13.09[bc]	1.23	13.87[b]	1.84	12.92[c]	1.65	5.47	3.73×10^{-3}
Zinc finger BED-type containing 4 (MENNQEPR)	ZBED4	8.81[a]	9.25	0[b]	0	0[b]	0	0[b]	0	5.46	3.73×10^{-3}
Transient receptor potential cation channel subfamily C member 4 associated protein (MAAAPAA)	TRPC4AP	5.98[a]	7.24	0[b]	0	0[b]	0	0[b]	0	5.43	3.73×10^{-3}
acid phosphatase or acid phosphatase, prostrate (MSAVPLP)	ACP3 or ACPP	11.82[a]	1.13	10.48[b]	1.05	110[b]	1.44	10.50[b]	2.03	5.43	3.73×10^{-3}

Protein name (peptide sequence)	Gene name	Control		Grade 1 FMC		Grade 2 FMC		Grade 3 FMC		-log 10c(p)	FDR
		Median	IQR	Median	IQR	Median	IQR	Median	IQR		
Serine/threonine-protein phosphatase (MSDSEKL)	PPP1CA	10.37[a]	1.70	0[b]	8.41	0[b]	4.40	0[b]	8.73	5.33	4.31×10^{-3}
Stomatin like 2 (MLARAAR)	STOML2	10.77[a]	1.29	0[b]	8.05	0[b]	11.26	0[b]	9.25	5.09	7×10^{-3}
HECT domain E3 ubiquitin protein ligase 3 (MAGPGPG)	HECTD3	13.64[a]	1.63	11.08[b]	3.33	12.93[b]	2.44	12.05[b]	2.18	50	7.09×10^{-3}
Ribonuclease L (MESKNHN)	RNASEL	10.23[a]	1.16	0[b]	0	7.82[b]	10.29	0[b]	8.54	50	7.09×10^{-3}
NAC-A/B domain-containing protein (MGSLSAA)	n/a	6.32[a]	7.29	0[b]	0	0[b]	0	0[b]	5.50	50	7.09×10^{-3}

Abbreviations: FMC, feline mammary carcinoma; IQR: interquartile range.

Statistical comparisons among groups were conducted using the Kruskal-Wallis test, with FDR values indicating significant differences. Different superscript letters denote significant differences between groups based on pairwise comparisons using the Mann-Whitney U test.
