# Supplementary material for: Phosphoproteomic profiling of feline mammary carcinoma: Insights into tumor grading and potential therapeutic targets
Source: PLoS One. 2025 Aug 21;20(8):e0330520. doi: 10.1371/journal.pone.0330520 (PMC12370146; doi:10.1371/journal.pone.0330520)
Supplement: S3 Table — (DOCX) [file pone.0330520.s004.docx]

**S3 Table. Comparison of 17 differential proteins expression among 5 molecular subtypes of feline mammary carcinoma.**

| **Protein name** | **Molecular subtype** | | | | | | | | | | ***p* value** |
| --- | --- | --- | --- | --- | --- | --- | --- | --- | --- | --- | --- |
|  | **Luminal B HER–** | | **Luminal B HER2+** | | **HER2+** | | **Triple negative/ basal-like** | | **Triple negative/ normal-like** | |  |
|  | **Median** | **IQR** | **Median** | **IQR** | **Median** | **IQR** | **Median** | **IQR** | **Median** | **IQR** |  |
| FBXO7 | 0.00 | 2.85 | 0.00 | 4.1 | 1.22 | 4.74 | 2.17 | 2.49 | 0.00 | 2.74 | 0.93 |
| NSD3 | 0.00 | 2.34 | 0.00 | 2.88 | 0.00 | 2.28 | 0.00 | 0.44 | 3.32 | 6.5 | 0.44 |
| NUDT12 | 0.00 | 0.00 | 0.00 | 0.00 | 0.00 | 0.00 | 0.00 | 1.4 | 0.00 | 0.00 | 0.38 |
| JAKMIP2 | 0.00 | 0.00 | 0.00 | 2.93 | 0.00 | 3.47 | 0.00 | 0.5 | 0.00 | 2.2 | 0.91 |
| BTN1A1 | 1.94 | 6.02 | 0.00 | 2.41 | 0.91 | 5.27 | 1.89 | 4.47 | 0.00 | 3.17 | 0.52 |
| LOC101097497 | 8.20 | 8.9 | 7.46 | 8.35 | 5.75 | 8.05 | 5.11 | 3.99 | 7.02 | 6.69 | 0.75 |
| ABCC3 | 0.00 | 2.72 | 0.00 | 1.22 | 1.23 | 3.84 | 0.00 | 1.92 | 2.02 | 3.87 | 0.43 |
| PRKAG3 | 0.00 | 2.22 | 0.00 | 3.62 | 2.03 | 6.72 | 0.83 | 2.54 | 0.00 | 3.88 | 0.71 |
| KRT10 | 14.20 | 2.34 | 12.44 | 1.38 | 13.42 | 1.61 | 13.32 | 1.33 | 12.37 | 2.49 | 0.33 |
| ZBED4 | 0.00 | 0.00 | 0.00 | 4.14 | 1.15 | 2.75 | 0.00 | 3.32 | 0.00 | 2.89 | 0.54 |
| TRPC4AP | 0.00 | 0.00 | 0.00 | 1.21 | 1.11 | 3.54 | 0.00 | 0.44 | 2.16 | 5.82 | 0.11 |
| ACP3 | 8.97 | 3.57 | 8.33 | 3.36 | 11.53 | 1.87 | 9.94 | 1.78 | 10.71 | 3.22 | 0.10 |
| PPP1CA | 1.47 | 5.74 | 0.00 | 8.23 | 1.40 | 5.85 | 2.40 | 5.65 | 0.00 | 7.42 | 0.94 |
| STOML2 | 0.00 | 9.61 | 1.34 | 10.87 | 0.00 | 8.95 | 0.00 | 3.28 | 2.68 | 8.63 | 0.81 |
| HECTD3 | 12.07 | 6.14 | 12.22 | 3.29 | 12.86 | 2.91 | 11.10 | 3.14 | 11.51 | 3.3 | 0.55 |
| RNASEL | 6.49 | 6.28 | 0.00 | 7.05 | 5.02 | 10.25 | 3.68 | 8.45 | 0.00 | 3.87 | 0.48 |
| NAC-A/B | 0.00 | 2.01 | 1.40 | 4.88 | 0.00 | 1.65 | 0.00 | 0.76 | 1.72 | 3.15 | 0.55 |

Statistical comparisons among groups were conducted using the Kruskal-Wallis test. The significant difference was considered when P < 0.05.
